# Therapeutic potential of hesperetin and isoliquiritigenin in chronic kidney disease

**DOI:** 10.17179/excli2026-9683

**Published:** 2026-09-11

**Authors:** Meenu Chhikara, Mohd Aqil, Syed Ehtaisham Ul Haque, Mohd Akhtar, Abul Kalam Najmi

**Affiliations:** 1 Department of Pharmacology, School of Pharmaceutical Education and Research, Jamia Hamdard, New Delhi 110062, India; 2 Department of Pharmaceutics, School of Pharmaceutical Education and Research, Jamia Hamdard, New Delhi 110062, India

## ⁯

Type 2 diabetes mellitus (T2DM) is a metabolism dysfunction with persistently elevated levels of glucose and resistance in insulin (Yang et al., 2020[6]). If the elevated blood glucose levels in T2DM are not effectively controlled, it can lead to chronic kidney disease (CKD) development which can further progress to end stage renal disease (ESRD) (Bhattacharjee et al., 2016[2]; Aboismaiel et al., 2024[1]).

The characteristic features of CKD include glomerulosclerosis, albuminuria, glomerulus hyperfiltration, and fibrotic changes. Inflammation has been found to play a significant role in CKD development (Bhattacharjee et al., 2016[2]; Habli, 2024[4]). Therefore, inhibition of inflammatory signals may protect the development of CKD in T2DM patients. Further, current treatment options are not adequate for the management of T2DM and its associated complications. Flavonoids are natural compounds known for their bioactive properties, especially in the modulation of oxidative and inflammatory pathways. Hesperetin (HSP) is one of the key flavanones found in citrus fruits while isoliquiritigenin (ISL), another flavonoid, is found in the Glycyrrhiza uralensis roots. Both HSP and ISL exhibit various therapeutic benefits due to their anti-inflammatory and antioxidant properties. Therefore, we have assessed the preclinical data on ISL and HSP's role in the kidney disease protection and their influence on the modulation of inflammatory mediators.

The assessment involved an extensive bibliographic search from PubMed, Google Scholar, and ScienceDirect. The keywords for the search included “hesperetin” OR “isoliquiritigenin” alone or with specific search terms such as “diabetes”, “nephropathy”, “kidney disease”, “renal injury” and “inflammation”. The potentially relevant literature was identified and summarized in this report. Additionally, random Google searches were performed for the relevant literature. The inclusion criteria included: a) studies investigating the pharmacological effects of HSP and ISL in animal models; b) research focusing on the role of HSP and ISL in kidney disease. The exclusion criteria were: a) purely theoretical studies with no preclinical application and human studies; b) studies not available in English.

HSP and ISL have diverse pharmacological effects. Animal pharmacological studies have shown HSP and ISL as the drugs in the protection of kidney disease via inhibition of inflammatory signaling pathways (Supplementary Table 1). In CKD, persistent hyperglycemia, along with insulin resistance and dyslipidemia, stimulates kidney inflammation by activation of inflammatory signaling pathways such as NF-κB (nuclear factor-κB) and NLRP3 (NLR family pyrin domain containing 3), that further upregulate the proinflammatory cytokines such as IL-6 (interleukin-6), IL-1β (interleukin-1β) and TNF-α (tumor necrosis factor-α) (Sahakyan et al., 2022[5]; Aboismaiel et al., 2024[1]). The inflammatory cytokines further recruit immune cells to renal tissue, leading to pathophysiological characteristics such as renal injury, mesangial expansion, and damage to glomerulus, ultimately resulting in CKD (Donate-Correa et al., 2021[3]). We observed that HSP and ISL showed a protective effect in acute and chronic kidney disease, which may be via the inhibition of cytokine levels, antioxidant effect and improvement of glycemic status in T2DM rats.

## Declaration

### Conflict of interest

The authors declare no conflict of interest.

### Funding

None.

### Artificial Intelligence (AI) - assisted technology

The authors did not use AI tools for data analysis, interpretation, or the generation of scientific conclusions. The authors assume full responsibility for the accuracy, originality, and scientific integrity of the final content of the published letter.

## Supplementary Material

Supplementary information
