# Supplementary material for: Therapeutic potential of hesperetin and isoliquiritigenin in chronic kidney disease
Source: EXCLI J. 2026 Sep 11;25:1486–7. doi: 10.17179/excli2026-9683 (PMC13731906; doi:10.17179/excli2026-9683)
Supplement: Supplementary file 1 [file EXCLI-25-1486-s-001.pdf]

EXCLI Journal 2026;25:1486-1487 – ISSN 1611-2156
Received: July 24, 2026, accepted: August 31, 2026, published: September 11, 2026

Supplementary information to:

Letter to the editor:

THERAPEUTIC POTENTIAL OF HESPERETIN AND ISOLIQUIRITIGENIN IN CHRONIC KIDNEY DISEASE

Meenu Chhikara[1], Mohd Aqil[2], Syed Ehtaisham Ul Haque[1], Mohd Akhtar[1], Abul Kalam Najmi[1*]

[1] Department of Pharmacology, School of Pharmaceutical Education and Research, Jamia Hamdard, New Delhi 110062, India
[2] Department of Pharmaceutics, School of Pharmaceutical Education and Research, Jamia Hamdard, New Delhi 110062, India

* **Corresponding author:** Dr. Abul Kalam Najmi, Department of Pharmacology, School of Pharmaceutical Education and Research, Jamia Hamdard, New Delhi 110062, India; E-mail: aknajmi@jamiahamdard.ac.in

https://dx.doi.org/10.17179/excli2026-9683

This is an Open Access article distributed under the terms of the Creative Commons Attribution License (https://creativecommons.org/licenses/by/4.0/).

Supplementary Table 1: Potential activities of hesperetin and isoliquiritigenin in animal models of kidney disease

Drug	Model	Key Findings	Inflammatory Markers Affected	Reference
**Hesperetin**	STZ-induced DN in Albino rats	Antioxidant, anti-inflammatory, antidiabetic, decrease GSK-3β expression and antiapoptosis	TGF-β, TNF-α, and IL-6	Abdou and Abd Elkader, 2022
**Hesperetin**	LPS-induced AKI in C57BL/6N	Antioxidant, antiapoptotic, and antiinflammatory	TNF-α, IL-6, and IL-1β	Yang et al., 2023
**Hesperetin**	HFD-STZ-induced DN in Wistar rats	Antioxidant, antiinflammatory, and antihyperglycemic	NLRP3, NF-κB, TNF-α, IL-6, and IL-1β	Meenu et al., 2025
**Hesperetin**	HFD-STZ-induced DN in Wistar rats	Antioxidant, antiinflammatory, and antihyperglycemic	TNF-α, IL-6, and IL-1β	Meenu et al., 2026
**Isoliquiritigenin**	STZ-induced DN in rats	Preserved renal architecture, restoration of Sirt1 and renal oxidant-antioxidant balance, dampening of inflammation and attenuation of collagen accumulation	NLRP3, NF-κB, IL-1β, TNF-α, and IL-10	Alzahrani et al., 2020
**Isoliquiritigenin**	STZ-induced renal injury in C57BL/6 mice	Antiinflammatory, antioxidant, and inhibition of MAPK activation, and the induction of Nrf-2 signaling	MCP-1 and IL-6	Huang et al., 2020
**Isoliquiritigenin**	UUO-induced renal inflammation and fibrosis in C57BL/6 mice	Antiinflammatory, antifibrotic, and suppression of mincle/Syk/NF-NF-κB signaling pathway	IL-1β, IL-6, TNF-α, iNOS, p-Syk, p-NF-κB, α-SMA and FN	Liao et al., 2020
**Isoliquiritigenin**	Cisplatin-induced AKI in C57BL/6 mice	Inhibition of FPR2 involved macrophagic inflammation	TNF-α, IL-6, and IL-1β	Rui-Zhi et al., 2022
**Isoliquiritigenin**	STZ-induced AKI in Sprague-Dawley rats	Antioxidant, antifibrosis, and inhibition of JAK2/STAT3 pathway	IL-6, ICAM-1, JAK2, p-JAK2, STAT-3, and p-STAT-3	Sun et al., 2021
**Isoliquiritigenin**	HFS and STZ-induced DKD in C57BL/6 mice	Antioxidant, antiinflammatory, inhibition of NF-κB and NLRP3 inflammasome pathway	NF-κB and NLRP3	Wang et al., 2024
**Isoliquiritigenin**	High glucose-induced GMC proliferation in mice	Antiproliferative, antiinflammatory, and inhibition of JAK2/STAT3 pathway	TNF-α, IL-1β, and TGF-β1	Zhang et al., 2024

AKI: Acute kidney injury; **DKD:** Diabetic kidney disease; **FN:** Fibronectin; **GSK-3β:** Glycogen synthase kinase-3 beta; **HFD:** High-fat diet; **HSP:** Hesperetin; **ICAM-1:** Intercellular adhesion molecule-1; **IL-1β:** Interleukin-1beta; **IL-6:** Interleukin-6; **IL-10:** Interleukin-10; **iNOS:** Inducible nitric oxide synthase; **ISL:** Isoliquiritigenin; **JAK2:** Janus kinase 2; **LPS:** Lipopolysaccharide; **MCP-1:** Monocyte chemoattractant protein-1; **NF-κB:** Nuclear factor κB; **NLRP3:** NLR family pyrin domain containing 3; **STAT-3:** Signal transducer and activator of transcription 3; **STZ:** Streptozotocin; **TGF-β1:** Transforming growth factor-beta1; **TNF-α:** Tumor necrosis factor-alpha; **UUO:** Unilateral ureteral obstruction; **α-SMA:** Alpha-smooth muscle actin

REFERENCES

Abdou HM, Abd Elkader HAE. The potential therapeutic effects of Trifolium alexandrinum extract, hesperetin and quercetin against diabetic nephropathy via attenuation of oxidative stress, inflammation, GSK-3β and apoptosis in male rats. Chem Biol Interact. 2022; 352:109781.
https://doi.org/10.1016/j.cbi.2021.109781

Alzahrani S, Zaitone SA, Said E, El-Sherbiny M, Ajwah S, Alsharif SY, et al. Protective effect of isoliquiritigenin on experimental diabetic nephropathy in rats: Impact on Sirt-1/NFκB balance and NLRP3 expression. Int Immunopharmacol. 2020;87:106813.
https://doi.org/10.1016/j.intimp.2020.106813

Huang X, Shi Y, Chen H, Le R, Gong X, Xu K, et al. Isoliquiritigenin prevents hyperglycemia-induced renal injuries by inhibiting inflammation and oxidative stress via SIRT1-dependent mechanism. Cell Death Dis. 2020;11:1040.
https://doi.org/10.1038/s41419-020-03260-9

Liao Y, Tan RZ, Li JC, Liu TT, Zhong X, Yan Y, et al. Isoliquiritigenin attenuates UUO-induced renal inflammation and fibrosis by inhibiting Mincle/Syk/NF-Kappa B signaling pathway. Drug Des Devel Ther. 2020;14:1455–68.
https://doi.org/10.2147/DDDT.S243420

Meenu, Bano U, Mujeeb M, Akhtar M, Aqil M, Haque SEU, et al. Hesperetin and isoliquiritigenin attenuate the progression of diabetic nephropathy via inhibition of NF-κB/NLRP3 pathways in type 2 diabetic rats. ACS Omega. 2025;10:34342–51.
https://doi.org/10.1021/acsomega.5c02026

Meenu, Aqil M, Haque SEU, Akhtar M, Mujeeb M, Zaidi Z, et al. Hesperetin and isoliquiritigenin ameliorates the hepatic and renal complications in type 2 diabetic rats. Journal of Pharmacology and Pharmacotherapeutics. 2026;0(0).
https://doi.org/10.1177/0976500X261463364

Rui-Zhi T, Ke-Huan X, Yuan L, Xiao L, Bing-Wen Z, Tong-Tong L, et al. Renoprotective effect of isoliquiritigenin on cisplatin-induced acute kidney injury through inhibition of FPR2 in macrophage. J Pharmacol Sci. 2022;148:56–64.
https://doi.org/10.1016/j.jphs.2021.10.001

Sun L, Yang Z, Zhang J, Wang J. Isoliquiritigenin attenuates acute renal injury through suppressing oxidative stress, fibrosis and JAK2/STAT3 pathway in streptozotocin-induced diabetic rats. Bioengineered. 2021; 12:11188–200.
https://doi.org/10.1080/21655979.2021.2006978

Wang Y, Yang J, Chang X, Xue Y, Liu G, Zhang T, et al. Isoliquiritigenin alleviates diabetic kidney disease via oxidative stress and the TLR4/NF-κB/NLRP3 inflammasome pathway. Mol Nutr Food Res. 2024;68: 2400215.
https://doi.org/10.1002/mnfr.202400215

Yang AY, Choi HJ, Kim K, Leem J. Antioxidant, antiapoptotic, and anti-inflammatory effects of hesperetin in a mouse model of lipopolysaccharide-induced acute kidney injury. Molecules. 2023;28:2759.
https://doi.org/10.3390/molecules28062759

Zhang Z, Deng S, Shi Q. Isoliquiritigenin attenuates high glucose-induced proliferation, inflammation, and extracellular matrix deposition in glomerular mesangial cells by suppressing JAK2/STAT3 pathway. Naunyn Schmiedebergs Arch Pharmacol. 2024;397: 123–31.
https://doi.org/10.1007/s00210-023-02598-z
